# Plasma Pharmacokinetic Profile of Fipronil 1% *Pour‐On* in Cattle: Photodegradation and the Sustained Systemic Persistence of Fipronil Sulfone

**DOI:** 10.1111/jvp.70085

**Published:** 2026-05-15

**Authors:** Stefani Maria Ferreira, Leonila Ester Reinert Raspantini, Giselle Kindlein, Fabiano Barreto, Caroline Andrade Tomaszewski, Tamara dos Santos Castilhos, Daniel Rodrigo Hillesheim, Silvana Lima Górniak, André Tadeu Gotardo

**Affiliations:** ^1^ Research Centre for Veterinary Toxicology (CEPTOX), Department of Pathology School of Veterinary Medicine and Animal Sciences, University of São Paulo Pirassununga SP Brazil; ^2^ Ministry of Agriculture and Livestock, Esplanada dos Ministérios Brasília DF Brazil; ^3^ Federal Agricultural Defense Laboratory (LFDA‐RS) Ministry of Agriculture and Livestock Porto Alegre RS Brazil

**Keywords:** *Bos indicus*, fipronil desulfinyl, marker residue, phenylpyrazole, safety assessments

## Abstract

This study characterized the long‐term pharmacokinetic profile of a 1% fipronil *pour‐on* formulation in Nellore bulls under field conditions. Seventeen animals received a single topical dose (1 mg/kg), and plasma concentrations of fipronil and its metabolites (sulfone and desulfinyl) were monitored for 175 days using LC–MS/MS. Fipronil was rapidly absorbed, reaching a maximum concentration *C*_max_ of 42.3 ± 4.1 ng/mL on day 1, but became undetectable by day 35. Conversely, fipronil sulfone was the predominant systemic analyte, reaching *C*_max_ 60.2 ± 5.6 ng/mL on day 13 and persisting for up to 140 days. The metabolite‐to‐parent AUC_0–*t*_ ratio (6.2) and the protracted terminal phase are consistent with flip‐flop kinetics, where systemic persistence is governed by slow release from the dermal/sebaceous reservoir rather than metabolic clearance. Fipronil desulfinyl was sporadically detected at low levels, confirming that in vivo photodegradation occurs on the animal's surface. These findings consolidate fipronil sulfone as the most reliable marker residue for food safety assessments. Furthermore, the sporadic detection of fipronil desulfinyl confirms that in vivo photodegradation occurs under field conditions, warranting further investigation regarding its contribution to residue dynamics in cattle.

## 1 Introduction

Fipronil [5‐amino‐3‐cyano‐1‐(2, 6‐dichloro 4 trifluoromethylphenyl)‐4 trifluoromethyl sulfinyl pyrazole], a broad‐spectrum phenylpyrazole pesticide, was first synthesized in 1987 (Tingle et al. 2003) and introduced commercially in 1993 (Tomlin 2000). Originally developed for agricultural use as a safer alternative to highly toxic organophosphorus compounds (Li et al. 2020), fipronil acts by disrupting the insect's central nervous system. Specifically, it antagonizes γ‐aminobutyric acid (GABA) receptors and glutamate‐gated chloride (GluCl) channels, thereby inhibiting chloride ion influx (Grant et al. 1998). This blockage leads to neuronal hyperexcitation, paralysis, and death of the target pest (Ohi et al. 2004). Notably, fipronil exhibits a markedly higher binding affinity for insect GABA receptor complexes than for mammalian ones (Zhao et al. 2004), a differential selectivity that provides a substantial safety margin for humans and non‐target animals.

Currently, fipronil exhibits dual‐use characteristics, being extensively employed worldwide as an agricultural insecticide for the control of a broad range of crop pests (Tingle et al. 2003; FAO/WHO 2022). In the veterinary field, however, its use remains largely restricted to companion animals in most countries, particularly in the United States and the European Union, where products containing fipronil are authorized for the control of fleas and ticks in dogs and cats (Yuskiv et al. 2024; Joachim et al. 2025). Nevertheless, in several regions of the world, including parts of Asia and the Middle East, fipronil has also been used for ectoparasite control in livestock under local veterinary practices (Tingle et al. 2003; Singh et al. 2021). This practice is particularly notable in some Latin American countries, where fipronil continues to be used in cattle as a topical ectoparasiticide (Lopes et al. 2017). In addition, off‐label use has been reported in small ruminants, particularly goats, mainly for the control of ectoparasites when species‐specific therapeutic options are limited (Corke and Matthews 2018).

The regulatory status of fipronil in livestock production varies considerably across the globe, reflecting the balance between its parasiticidal efficacy and concerns related to food safety. In major bovine beef‐exporting countries such as Brazil, Argentina, South Africa, and India, fipronil remains an important tool for controlling ectoparasites, including populations resistant to other parasiticides, and formulations for bovine use remain registered.

While foundational metabolic pathways for fipronil were initially described in regulatory risk assessment reports and unpublished dossiers (AFSSA and AFSSE 2005; EFSA 2012; FAO/WHO 2022), the pharmacological landscape has been significantly expanded by recent peer‐reviewed research. Several studies have characterized the pharmacokinetics of fipronil and its sulfone metabolite across diverse species and formulations, providing insights into its systemic disposition and toxicological profile (Cid et al. 2016; Chata et al. 2019; Chang and Tsai 2020; Santos et al. 2022; Rocha et al. 2026; Ferreira et al. 2026). Despite these advancements, there is a notable scarcity of standardized data detailing the long‐term depletion kinetics of fipronil, and particularly its metabolite, fipronil sulfone, under representative field conditions in cattle. Understanding these dynamics is essential, as the high lipophilicity of these compounds leads to extensive tissue distribution and preferential accumulation in adipose tissue due to its high lipophilicity.

Following absorption, the compound undergoes extensive hepatic biotransformation. The principal metabolites identified include fipronil sulfone, formed through oxidative metabolism mediated by cytochrome P450 enzymes, as well as fipronil sulfide and fipronil desulfinyl, which may arise through reductive or photolytic pathways (Hainzl and Casida 1996; Tang et al. 2004; FAO/WHO 2022). While fipronil sulfone has been the metabolite most extensively investigated in animal studies, fipronil desulfinyl is primarily described as a photodegradation product formed after exposure of fipronil to ultraviolet radiation, with most available data originating from environmental and plant metabolism studies rather than from investigations in livestock (Ying and Kookana 2002; Thuyet et al. 2011). Excretion occurs primarily via the biliary–fecal route, with evidence of enterohepatic recirculation. Furthermore, the potential for transfer into milk remains a critical concern for food safety (Mohamed et al. 2004; EFSA 2012; FAO/WHO 2022).

The pharmacokinetics of fipronil are also strongly influenced by the route of administration and by its lipophilic properties. When administered via topical (*pour‐on*) formulations, the compound distributes mainly within the stratum corneum, epidermis, and pilosebaceous units. Within these structures, fipronil is preferentially sequestered in the sebaceous glands and around the hair follicles, from which it is slowly released through the follicular ducts (Birckel et al. 1998). This gradual and prolonged release is attributed to a process known as translocation, involving passive diffusion through the sebaceous secretions across the skin surface and pelage (Tanner et al. 1997), a mechanism that enables an extended period of protection against ectoparasite reinfestation.

However, this extensive distribution across the animal's coat inherently exposes the compound to environmental factors. Given that fipronil is highly susceptible to photodegradation, the formation of fipronil desulfinyl, its principal photometabolite (Pei et al. 2004), becomes an important experimental consideration in topical treatments. Under field conditions with direct solar radiation, such transformation may occur on the hair coat and skin surface following *pour‐on* administration. Therefore, evaluating this metabolite was essential to assess the environmental stability of the formulation and to avoid misinterpretation of the pharmacokinetic behavior of the active compound. In addition, given its high lipophilicity, investigating whether this photometabolite may undergo systemic absorption could help clarify its potential relevance as a marker residue in food safety assessments.

Despite the critical need for such comprehensive safety and kinetic data, most available pharmacokinetic parameters are extrapolated from non‐ruminant models or restricted to internal regulatory dossiers. Peer‐reviewed studies detailing the specific depletion kinetics, especially under representative field conditions, are notably scarce. To address this critical gap in pharmacological knowledge, the present study aimed to characterize the pharmacokinetic profile of a commercial 1% fipronil formulation administered via the topical (*pour‐on*) route in cattle.

## 2 Material and Methods

This investigation was conducted in strict accordance with the guidelines of the International Cooperation on Harmonisation of Technical Requirements for Registration of Veterinary Medicinal Products (VICH GL46 2012), which advocate for metabolism studies utilizing the intended route of administration. Consequently, these findings provide robust, standardized data essential for providing a scientific basis that supports regulatory decisions, contributing to the harmonization of international trade, and improving the framework for food safety assessments.

All experimental procedures were approved by the Ethics Committee on the Use of Animals (CEUA) of the School of Veterinary Medicine and Animal Science, University of São Paulo (FMVZ‐USP; protocol number 7756291123). Animal care and handling were performed by experienced personnel under constant veterinary supervision, ensuring adherence to high welfare standards. The in vivo phase of the study, including drug administration and serial blood sampling, was conducted between July 2024 and January 2025 at the Veterinary Toxicology Research Center (CEPTOX) located in the “Fernando Costa” campus of the University of São Paulo (USP) in Pirassununga, São Paulo, Brazil (21°59′46.00″ S, 47°25′32.99″ W).

### 2.1 Animals, Feeding, and Housing

Seventeen Nelore bulls (*Bos indicus indicus*), approximately 10 months old with a mean body weight (BW) of 292.3 ± 3.4 kg, were used. The animals were representative of the target category for fipronil application, aligning with the VICH GL46 guidelines (2012). The cattle were maintained on a pasture of *Brachiaria* spp., with ad libitum access to water and mineral supplements. To ensure no prior chemical interference, the pasture had no history of fipronil application, and none of the animals had been exposed to phenylpyrazole‐based drugs for at least 180 days preceding the trial.

Prior to the experimental phase, a comprehensive clinical screening was conducted to confirm the animals' health status, physiological condition, and body condition score. Each animal underwent visual inspection for ectoparasites (ticks, flies, and warbles) and was weighed using a hydraulic squeeze chute equipped with a calibrated electronic scale.

Following the initial screening, blood samples were collected via jugular venipuncture for hematological and biochemical analyses. Hematological parameters including red blood cell count, hemoglobin, hematocrit, mean corpuscular volume (MCV), mean corpuscular hemoglobin (MCH), mean corpuscular hemoglobin concentration (MCHC), and total/differential leukocyte counts were determined according to the protocols of the Clinical Analysis Laboratory at the CEPTOX. Serum biochemical analyses were performed using an automated ChemWell‐T analyzer (Labtest) and commercial Bioclin kits to quantify albumin, total protein, cholesterol, glucose, urea, creatinine, and the enzymatic activities of aspartate aminotransferase (AST), alanine aminotransferase (ALT), gamma‐glutamyl transferase (GGT), and alkaline phosphatase (ALP). Additionally, gastrointestinal parasite burden was monitored via fecal egg counts (McMaster technique), complemented by visual ectoparasite inspections. To ensure continued health during this long‐term study, these clinical and laboratory evaluations were repeated at mid‐point (day 63) and end‐point (day 175) of the experimental period.

### 2.2 Treatment and Sampling

A commercial 1% Fipronil *pour‐on* formulation (Topline, Boehringer Ingelheim) was used in this study. The product was administered topically at the label recommended dose of 1 mg/kg BW (1 mL per 10 kg BW). To ensure maximum dosing accuracy, the required volume for each animal was measured using a 60 mL graduated syringe and applied along the dorsal midline, extending from the first thoracic to the last lumbar vertebrae. Administration was performed for all animals in a single session, between 08:00 and 09:00 a.m.

Baseline blood samples (Day 0) were collected via jugular venipuncture into heparinized tubes 15 min prior to drug administration. Following treatment, serial blood samples were collected on days 1, 3, 6, 9, 13, 16, 22, 28, 35, 49, 63, 77, 91, 105, 119, 140, and 175 post‐administrations. Immediately after collection, blood samples were centrifuged at 3000 *g* for 15 min. To ensure analyte integrity, plasma samples were harvested and stored at −20°C and analyzed in batches within 90 days of collection. This analytical window was strictly maintained to comply with the established 3‐month stability period for fipronil and its metabolites in frozen bovine plasma, as previously demonstrated by Cid et al. (2012).

### 2.3 Analytical Procedures

Sample analyses were conducted at the Federal Laboratory for Agricultural Defense of Rio Grande do Sul (LFDA/RS), in Porto Alegre, Brazil, a facility affiliated with the Ministry of Agriculture and Livestock (MAPA). The extraction and quantification of fipronil, fipronil sulfone, and fipronil desulfinyl followed the method described by Rubensam et al. (2013), with specific adaptations and validation according to the MAPA Analytical Quality Assurance Manual (Brasil 2015).

#### 2.3.1 Chemicals and Reagents

High‐purity analytical standards (> 97%) of fipronil and fipronil sulfone were sourced from Dr. Ehrenstorfer (LGC Standards, Germany), while the fipronil desulfinyl standard (PESTANAL; purity > 94%) was acquired from Sigma‐Aldrich Production GmbH (Switzerland). Acetonitrile and acetic acid (HPLC grade) were obtained from J. T. Baker (USA), and ammonium acetate was sourced from Mallinckrodt‐Baker (USA). Individual stock solutions (1.0 mg/mL) were prepared by dissolving each standard in acetonitrile. Working solutions were prepared by serial dilution to establish a 9‐point calibration curve, covering a linear range of 0.5–100 ng/mL for fipronil, fipronil sulfone and fipronil desulfinyl. All standard solutions were stored in polypropylene tubes at −20°C.

#### 2.3.2 Sample Preparation and Extraction

The analytes were extracted from plasma via protein precipitation. Briefly, 300 μL plasma aliquots were treated with sequential additions of acetonitrile to optimize extraction efficiency and minimize matrix effects. Three successive 100 μL aliquots of acetonitrile were added, followed by a final 300 μL volume, with vigorous vortexing for 15 s after each step. The mixture was then centrifuged at 12,000 *g* for 10 min at 5°C to facilitate phase separation. Finally, 700 μL of the supernatant was harvested and transferred to amber vials for LC–MS/MS analysis.

#### 2.3.3 LC–MS/MS Conditions

Chromatographic separation was performed using an Agilent 1260 Infinity II system (Santa Clara, CA, USA) equipped with a quaternary pump, degasser, autosampler, and column oven. Chromatographic separation was achieved on a Phenomenex Luna C18 column (50 mm × 2 mm i.d., 3 μm), preceded by a guard column (4 mm × 3 mm i.d.) of the same material (Torrance, CA, USA). The mobile phase consisted of water (A) and acetonitrile (B) at a flow rate of 0.5 mL min^−1^ using a gradient program: initial 15% B, increasing to 100% B over 2 min, held at 100% B until 5 min, returning to initial conditions at 5.5 min, and maintained until 7 min. The equilibration time between injections was 1 min. The injection volume was 2 μL, and the column temperature was maintained at 40°C.

The LC system was coupled to an Applied Biosystems API 5000 triple quadrupole mass spectrometer (Foster City, CA, USA) with an electrospray ionization (ESI) source operating in negative mode. Optimized source‐dependent parameters included an ion spray voltage of −4500 V, source temperature of 400°C, and gas settings for collision (CAD), nebulizer (GS1), and drying (GS2) at 8 psi, 50 psi, and 50 psi, respectively. Multiple Reaction Monitoring (MRM) was employed with a dwell time of 100 ms per transition. For fipronil, the quantification and confirmation transitions were *m/z* 435.13 → 250.1 (DP ‐70 V; CE ‐38 V; EP ‐10 V; CXP ‐15 V) and 435.13 → 278 (DP ‐70 V; CE ‐40 V; EP ‐10 V; CXP ‐15 V). Fipronil sulfone transitions were *m/z* 450.872 → 245.991 (DP ‐125 V; CE ‐52 V; EP ‐10 V; CXP ‐11 V) and 450.872 → 414.962 (DP ‐125 V; CE ‐37 V; EP ‐10 V; CXP ‐15 V). Finally, for fipronil desulfinyl, the transitions utilized were *m/z* 387.009 → 351.00 (DP ‐125 V; CE ‐23 V; EP ‐10 V; CXP ‐18 V) for quantification and 387.009 → 236.991 (DP ‐125 V; CE ‐54 V; EP ‐10 V; CXP ‐23 V) for confirmation.

#### 2.3.4 Method Validation

The analytical method was validated in accordance with the MAPA Analytical Quality Assurance Manual (Brasil 2015) and International Council for Harmonization (ICH) guidelines (2022). Selectivity was confirmed by the absence of endogenous interferences at the retention times of the analytes. Quantification was performed using external standardization with matrix‐matched calibration curves, prepared by fortifying blank bovine plasma to compensate for potential matrix effects. Linearity was evaluated over the working range of 0.5–100 ng/mL for fipronil and fipronil sulfone, and 1.0–100 ng/mL for fipronil desulfinyl, with coefficients of determination (*r* ^2^) exceeding 0.99 for all compounds. The LOQ was defined as the lowest point of the calibration curve with a signal‐to‐noise ratio ≥ 10. Precision and accuracy were considered acceptable with a coefficient of variation ≤ 15% and trueness within 85%–115%, respectively.

### 2.4 Pharmacokinetic Analysis

Pharmacokinetic (PK) parameters for fipronil and its metabolites were determined for each animal using a non‐compartmental analysis (NCA) using the PKSolver add‐in for Microsoft Excel (Zhang et al. 2010). Due to the homogeneity of the experimental group, which consisted exclusively of male cattle of similar age and weight, descriptive statistics were employed to characterize the population. Data expressed as mean ± standard error of the mean (SEM) was used to characterize the population and inter‐individual variability. The maximum plasma concentration (*C*_max_) and the time to reach it (*T*_max_) were obtained directly from the individual concentration‐time profiles. The area under the plasma concentration‐time curve from time zero to the last measurable concentration (AUC_0–*t*_) was calculated using the linear trapezoidal rule. The terminal elimination rate constant (Kel) was estimated by linear regression of the terminal phase of the semi‐logarithmic concentration‐time curve, and the terminal elimination half‐life (*t*_1/2z_) was calculated as *t*_1/2z_ = ln (2) / Kel.

Given that fipronil was administered via an extravascular route (*pour‐on*) and the absolute bioavailability (*F*) was unknown, all related parameters are reported as apparent values. Apparent systemic clearance (Cl/*F*) was calculated as Dose/AUC_0t_, and the apparent terminal volume of distribution (*V*_z_/*F*) was determined as (Cl/*F*)/Kel (Ye and Ducharme 2016).

## 3 Results

The analytical method demonstrated high selectivity, with no endogenous interferences observed at the retention times of fipronil, fipronil sulfone, or fipronil desulfinyl. Linearity was confirmed over the established working ranges, with *r* ^2^ exceeding 0.99 for all analytes. The LOQ was 0.5 ng/mL for fipronil and fipronil sulfone, and 1.0 ng/mL for fipronil desulfinyl. Intra‐day precision was high, with CV values below 7.1%, 5.6%, and 5.3% for fipronil and its metabolites, respectively. Accuracy (trueness) remained within the 85%–115% acceptance range, with values reaching up to 114%, 112%, and 106%, respectively (Table 1).

**TABLE 1:** Validation parameters for the determination of fipronil and its metabolites in bovine plasma by LC–MS/MS.

Validation parameters	Reference value	Fipronil	Fipronil sulfone	Fipronil desulfinyl
Selectivity	> 20% of LOQ	Complies	Complies	Complies
Linearity (*r* ^2^)	≥ 0.99	≥ 0.99	≥ 0.99	≥ 0.99
Intra‐day precision (CV, %)	≤ 15%	7.1	5.6	5.3
Accuracy (Trueness, %)	85%–115%	114	112	106
LOQ (ng/mL)	S/N ≥ 10	0.5	0.5	1

Abbreviations: CV, coefficient of variation; LOQ, limit of quantification; *r* ^2^, coefficient of determination.

No adverse clinical signs, localized skin reactions at the application site, or behavioral changes were observed in any of the animals throughout the 175‐day experimental period. The longitudinal monitoring of health status confirmed the stability of the biological model, as all hematological and biochemical parameters remained within the physiological reference ranges for the bovine species (Fighera 2023; Table 2). Parasitological assessment confirmed that the animals maintained a high health status under field conditions. Coproparasitological evaluations indicated a low level of endoparasite infestation throughout the study (Hassum 2008). Furthermore, visual inspections for ectoparasites consistently showed counts below the economic injury levels, with fewer than 20 ticks ( *Rhipicephalus microplus* ), 10 larvae (*Dermatobia hominis*), and 200 flies ( *Haematobia irritans* ) per animal (EMBRAPA 2017; Table 1). These results demonstrate that the animals remained healthy and relatively free of parasitic stress, ensuring that the subsequent pharmacokinetic data reflect the drug's behavior in a standardized physiological state.

**TABLE 2:** Clinical health markers, parasitological status, hematological and biochemical parameters of Nellore bulls treated with a veterinary formulation containing 1% fipronil (1 mg/kg, *pour‐on*; *N* = 17/time point; mean ± SEM).

Parameters	Reference value	Experimental days		
		0	63	175
Weight (kg)		292.3 ± 3.4	302.4 ± 3.4	351.2 ± 4.4
EPG	≥ 300	238.2 ± 47.5	270.6 ± 32.4	97.7 ± 20.6
Ectoparasites				
Tick	< 20	< 20	< 10	< 10
Larva	< 10	< 10	< 20	< 20
Fly	< 200	< 200	< 200	< 200
Hematological parameters				
Leukocytes (10^3^/mm^3^)	4.0–12.0	11.8 ± 0.4	10.5 ± 0.5	13.1 ± 0.7
Neutrophils (10^3^/mm^3^)	0.6–4.0	1.4 ± 0.2	2.6 ± 0.1	2.7 ± 0.3
Lymphocytes (10^3^/mm^3^)	2.5–7.5	9.7 ± 0.4	6.7 ± 0.5	8.8 ± 0.6
Monocytes (10^3^/mm^3^)	0.02–0.84	0.3 ± 0.0	0.5 ± 0.5	0.4 ± 0.0
Eosinophils (10^3^/mm^3^)	0–2.4	0.3 ± 0.2	0.7 ± 0.0	1.1 ± 0.1
Basophils (10^3^/mm^3^)	0–0.2	0.0 ± 0.0	0 ± 0	0 ± 0
Erythrocytes (×10^6^/mm^3^)	5–10	7.4 ± 0.5	9.6 ± 0.5	8.8 ± 0.3
Hemoglobin (g/dL)	8–15	13.4 ± 0.3	11.8 ± 0.4	11.5 ± 0.3
MCHC (g/dL)	30–36	36.7 ± 1.3	33.7 ± 0.8	38.2 ± 0.7
MCH (pg)	14.4–18.6	19.0 ± 0.0	12.7 ± 0.6	13.2 ± 0.4
MCV (fL)	40–60	51.7 ± 1.4	38 ± 2.1	34.5 ± 1.0
Hematócrito (%)	24–46	37.2 ± 1.4	35.1 ± 1.0	30.1 ± 0.6
Biochemical parameters				
GGT (μL/L)	6.1–17.4	14.8 ± 0.8	14.7 ± 1.0	20.8 ± 1.3
AST (μL/L)	78–132	52.6 ± 2.6	60.6 ± 2.3	92.9 ± 2.7
ALT (μL/L)	11–40	27.8 ± 1.6	30.7 ± 1.8	28.9 ± 1.4
ALP (μL/L)	0–488	294.5 ± 15.5	332.2 ± 23.7	287.4 ± 18.4
Glucose (mg/dL)	45–75	81.5 ± 2.9	74.8 ± 3.9	63.4 ± 2.6
Total protein (g/dL)	6.7–7.4	6.1 ± 0.1	5.7 ± 0.1	5.6 ± 0.1
Cholesterol (mg/dL)	80–120	129.2 ± 10.4	127.3 ± 8.5	109.0 ± 4.2
Albumin (g/dL)	3.0–3.5	3.2 ± 0.0	3.1 ± 0.0	3.39 ± 0.1
Urea (mg/dL)	20–30	19.5 ± 1.5	14.3 ± 0.9	26.1 ± 1.6
Creatinine (mg/dL)	1–2	2.8 ± 0.9	2.3 ± 0.1	1.6 ± 0.0

Abbreviations: ALP, alkaline phosphatase; ALT, alanine aminotransferase; AST, aspartate aminotransferase; EPG, eggs per gram; GGT, gamma‐glutamyl transferase; MCH, mean corpuscular hemoglobin; MCHC, mean corpuscular hemoglobin concentration; MCV, mean corpuscular volume.

**FIGURE 1:**
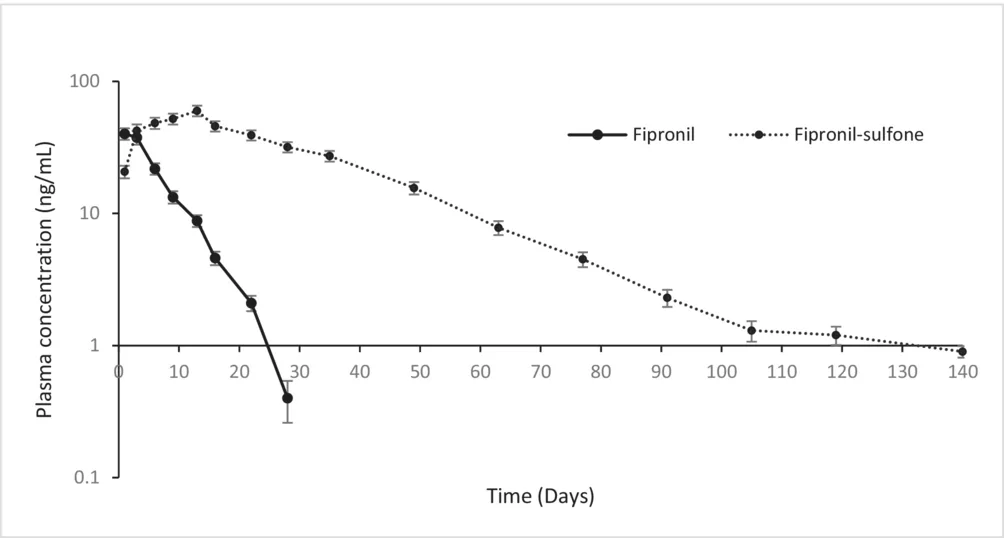
Mean ± SEM plasma concentration time profiles of fipronil and its metabolite fipronil sulfone in Nellore bulls after a single *pour‐on* administration of a veterinary formulation containing 1% fipronil (1 mg/kg; *n* = 17). Data are displayed using a semi‐logarithmic representation, with concentration on a logarithmic *Y*‐axis and days on a linear *X*‐axis.

The plasma concentration‐time profiles for fipronil and fipronil sulfone are summarized in Table 3, while the calculated pharmacokinetic parameters are presented in Table 4. The mean plasma concentration‐time curves are illustrated in Figure 1. On Day 0 (pre‐administration), no quantifiable levels of fipronil or its metabolites were detected in any of the animals. Following a single topical dose of 1 mg/kg, fipronil was rapidly absorbed, reaching its maximum plasma concentration (*C*_max_) of 42.3 ± 4.1 ng/mL on Day 1. After this initial peak, plasma levels declined progressively, reaching 13.3 ± 1.4 ng/mL on Day 9 and falling below the LOQ by Day 35.

**TABLE 3:** Mean followed by standard error of plasma concentrations of fipronil and fipronil sulfone (ng/mL) in Nellore bulls following a single topical administration of a veterinary formulation containing 1% fipronil (1 mg/kg, *pour‐on*; *N* = 17/time point; mean ± SEM).

Day	Fipronil (ng/mL)	Fipronil sulfone (ng/mL)
0	0 ± 0	0 ± 0
1	40.1 ± 4	20.7 ± 2.2
3	37.6 ± 4.5	42.3 ± 4.9
6	21.8 ± 2.1	48.3 ± 4.7
9	13.3 ± 1.4	52.0 ± 4.9
13	8.8 ± 0.9	59.9 ± 5.5
16	4.6 ± 0.5	45.8 ± 4.1
22	2.1 ± 0.2	39.1 ± 3.4
28	0.4 ± 0.1	31.8 ± 2.8
35	< LOQ	27.2 ± 2.5
49	< LOQ	15.6 ± 1.7
63	< LOQ	7.8 ± 0.9
77	< LOQ	4.5 ± 0.6
91	< LOQ	2.3 ± 0.3
105	< LOQ	1.3 ± 0.2
119	< LOQ	1.2 ± 0.2
140	< LOQ	0.9 ± 0.1
175	< LOQ	< LOQ

**TABLE 4:** Apparent pharmacokinetic parameters of fipronil and fipronil sulfone in Nellore bulls following a single topical administration of a veterinary formulation containing 1% fipronil (1 mg/kg, *pour‐on*; *N* = 17/time point; mean ± SEM).

Kinetic parameters	Fipronil	Fipronil sulfone
*C*_max_ (ng/mL)	42.3 ± 4.1	60.2 ± 5.6
*T*_max_ (days)	1.6 ± 0.2	12.7 ± 0.2
AUC_0–*t*_ (ng dia/mL)	333.1 ± 34.4	2070.8 ± 187.4
*t*_1/2z_ (days)	4.5 ± 0.1	20.8 ± 3.8
Kel (day^−1^)	0.2 ± 0	0.03 ± 0
Cl/*F* (L/day/kg)	3.58 ± 0.4	—
*V*_z_/*F* (L/kg)	23.1 ± 2.5	—

Abbreviations: AUC_0–*t*_, area under the plasma concentration–time curve from time zero to the last measurable concentration; Cl/F, apparent systemic clearance; *C*_max_, peak plasma concentration; *F*, fraction of the dose absorbed (bioavailability); Kel, terminal elimination rate constant; *t*_*1*/*2z*_, terminal elimination half‐life; *T*_max_, time of peak plasma concentration; *V*_*z*_/*F*, apparent terminal volume of distribution.

Fipronil sulfone was detected at the first sampling point (20.68 ± 2.2 ng/mL) and increased gradually to reach a *C*_max_ of 60.2 ± 5.6 ng/mL on Day 13. The metabolite demonstrated high persistence, with concentrations remaining quantifiable (0.2 ± 0.1 ng/mL) up to Day 140. By Day 3, the concentrations of fipronil sulfone had already surpassed those of the parent drug, remaining the predominant systemic marker until the end of the study. The resulting metabolite‐to‐parent AUC_0–*t*_ ratio was approximately 6.2, and the terminal elimination phase was notably protracted, suggesting that the elimination rate is limited by slow systemic absorption from the dermal reservoir (flip‐flop effect).

Regarding the photometabolite fipronil desulfinyl, concentrations remained below the LOQ (1.0 ng/mL) for the majority of the study population. Quantifiable levels were detected in only two animals at sporadic time points: the first animal showed concentrations of 1.05 and 1.76 ng/mL on Days 3 and 28, respectively, while the second animal presented a concentration of 1.17 ng/mL on Day 28. Due to this localized and infrequent detection, fipronil desulfinyl was excluded from the formal pharmacokinetic parameter estimation.

## 4 Discussion

The clinical and regulatory significance of veterinary pharmacokinetics extends far beyond determining drug efficacy; it constitutes a fundamental component of the scientific basis supporting the safety of animal‐derived food products (Toutain and Lees 2004; VICH GL48 2015). By elucidating the absorption, distribution, metabolism, and excretion of active substances, these studies map the disposition of parent drugs and their derivatives within the host (Aguilera‐Luiz et al. 2008; Nasim et al. 2016; Fahim et al. 2018; Canton et al. 2021). Such data are vital for identifying persistent metabolites that may sequester within edible tissues, a process that is fundamentally indispensable for the evidence‐based establishment of marker residues. This pharmacokinetic framework provides essential data for regulatory authorities to identify marker residues and design depletion studies necessary to define Maximum Residue Limits (MRLs), safeguarding public health and facilitating international trade (Sanders et al. 2016; Canton et al. 2021).

To ensure data reliability, animal‐related variables were controlled, as animal physiology significantly modulates pharmacokinetic profiles (Modric and Martinez 2011). All cattle underwent a selection process, including clinical, hematological, and biochemical screenings to rule out hepatic or renal dysfunctions that could impair drug clearance (Lea‐Henry et al. 2018). Plasma protein levels remained within physiological ranges throughout the 175‐day study; this stability served as a key indicator of the animals' adequate nutritional status and overall health, ensuring consistent drug binding and distribution kinetics (Kaneko et al. 2008; Wanat 2020). Since skin integrity and peripheral blood flow are critical for the absorption of *pour‐on* formulations (Magnusson et al. 2001), the absence of significant ectoparasite infestations was determinant. By minimizing parasitic stress and localized inflammation, factors known to alter protein binding and tissue perfusion (Jalali and Zeitlinger 2020), the observed kinetics can be confidently attributed to the formulation's intrinsic performance rather than host‐induced variability.

Environmental conditions were monitored to assess potential external influences on drug absorption. Meteorological records indicated stable ambient temperatures (~20°C) and no precipitation during the 30‐day post‐administration period. Because rainfall shortly after topical application can remove the product from the skin surface and compromise systemic uptake (Altenbrunner‐Martinek et al. 2020), the absence of precipitation likely favored drug retention and consistent transdermal absorption. Therefore, climatic conditions were unlikely to have significantly influenced the pharmacokinetic profiles observed in this study.

The pharmacokinetic profile of fipronil is multifactorial, dictated by the interplay between the route of administration, dose, and pharmaceutical formulation (Karademir et al. 2016). To the best of our knowledge, only two published studies have characterized fipronil plasma levels in cattle: one evaluating subcutaneous (SC) administration (Cid et al. 2016) and another investigating a *pour‐on* formulation combining fipronil and fluazuron (Lopes et al. 2017).

The substantial divergence between these reports and our findings underscores how different delivery strategies fundamentally reshape absorption kinetics, specifically by modulating the rate (*T*_max_) and extent (*C*_max_) of systemic exposure. In this sense, the study by Cid et al. (2016) using SC administration (1 mg/kg) in Zebu cattle reached a *C*_max_ of 378.1 ng/mL and a *T*_max_ of 10 h. In contrast, topical administration at the same dose in the present study resulted in a significantly lower *C*_max_ (42.3 ng/mL) and a delayed *T*_max_ (38.4 h). The SC route favored rapid systemic entry, yielding an AUC_0–*t*_ (19,388.6 ng day/mL) nearly 60 times higher than our topical AUC_0–*t*_ (333.1 ng day/mL). These differences stem from the physical barriers inherent to each route. While SC injection deposits the drug directly into the SC tissue (Durif et al. 1994), *pour‐on* absorption is restricted by skin thickness, hair follicle density, and local perfusion (Birckel et al. 1998). Furthermore, the topical “reservoir effect” (Tanner et al. 1997) which sequesters the drug in superficial layers and sebaceous glands explains the maintenance of lower, yet detectable, concentrations for up to 28 days.

Comparing our results with Lopes et al. (2017), who applied a fipronil‐fluazuron *pour‐on*, reveals that while their 25% higher dose explains their higher *C*_max_ (73.7 ng/mL), their substantially elevated *T*_max_ (60 h), AUC_0–*t*_ (36,699.4 ng day/mL), and *t*_1/2z_ (17 days) suggest a slower biotransformation. This highlights how concomitant active ingredients may alter the pharmacokinetic behavior of fipronil (Sánchez et al. 2002; Abo‐EL‐Sooud and Al‐Anati 2011). Furthermore, subspecies differences may also contribute to observed variability, as the use of purebred *Bos indicus* in the present study, compared to crossbred animals in Lopes et al. (2017), may influence drug disposition as previously documented for other antiparasitics such as ivermectin (Gotardo et al. 2020).

Regarding the metabolic profile, it is noteworthy that previous investigations of fipronil in cattle (Cid et al. 2016; Lopes et al. 2017) did not evaluate the plasma levels of its metabolites. In the present study, fipronil sulfone was the predominant analyte detected in the systemic circulation, exhibiting an AUC_0–*t*_ approximately 9.5‐fold higher than that of the parent compound. This predominance confirms that, once fipronil is absorbed, it undergoes intensive hepatic oxidation (Tang et al. 2004; EFSA 2012). This process is primarily mediated by the cytochrome P450 system, which efficiently converts fipronil into its sulfone derivative, a metabolite known for its higher stability and slower Cl/F compared to the parent drug (Leighait et al. 2009; FAO/WHO 2022).

The most notable pharmacokinetic finding observed in the present study was the prolonged persistence of fipronil sulfone, which remained quantifiable for up to 140 days. This extended detection window may not necessarily result from slow metabolic elimination; rather, it could represent a classic manifestation of the flip‐flop effect. In this phenomenon, the rate of drug absorption from the dermal reservoir is significantly slower than its rate of elimination (Toutain and Lees 2004). Consequently, the terminal slope of the plasma concentration‐time curve is governed by the slow, continuous release of fipronil from the skin and SC fat into the bloodstream, rather than the intrinsic capacity of the liver to clear the metabolite (Bousquet‐Mélou et al. 2004). This flip‐flop kinetics explains the long‐lasting residual efficacy of the *pour‐on* formulation against ectoparasites. However, from a food safety perspective, these findings suggest the potential for extended withdrawal periods, as the skin acts as a long‐term reservoir that maintains systemic levels of fipronil sulfone far beyond the initial application phase.

The minimal detection of fipronil desulfinyl in the plasma provides significant insights into the in vivo stability of the investigated *pour‐on* formulation. Although fipronil is highly susceptible to ultraviolet degradation, the fact that only two animals presented sporadic concentrations slightly above the LOQ suggests that the parent compound was rapidly sequestered into deeper cutaneous structures. This aligns with the translocation into pilosebaceous units, where fipronil is stored within sebaceous glands and protected from direct environmental exposure (Birckel et al. 1998). Probably, this biological reservoir effect, enhanced by photoprotective excipients in the formulation, ensures that the primary pathway for fipronil depletion is systemic absorption and hepatic metabolism into fipronil sulfone rather than external photolysis.

However, the sporadic detection of fipronil desulfinyl confirms that photodegradation on the animal's surface may occur under field conditions and leads to the systemic absorption of this active photometabolite. This finding is particularly relevant as fipronil desulfinyl is more lipophilic than the parent drug and has been associated with higher toxicity in certain models (Hainzl et al. 1998). While concentrations were low and not present in all animals, these results highlight a traditionally overlooked aspect of fipronil pharmacokinetics with significant implications for food safety. Given its high lipophilicity (Hainzl et al. 1998; Caboni et al. 2003), the potential for fipronil desulfinyl to exhibit distinct tissue sequestration dynamics warrants further investigation. While current regulatory frameworks focus on fipronil and fipronil sulfone as the primary marker residues (FAO/WHO 2021; Brasil 2022), the systemic presence of this photometabolite suggests that its role in long‐term depletion studies should be considered.

In conclusion, topical administration of fipronil in *Bos indicus* cattle resulted in limited systemic exposure to the parent compound and predominant formation of fipronil sulfone. The prolonged persistence of this metabolite, which remains detectable for up to 140 days, is consistent with flip‐flop kinetics driven by dermal reservoir formation after *pour‐on* application. These findings support the relevance of fipronil sulfone as the principal marker residue for regulatory monitoring of *pour‐on* formulations. The occasional detection of fipronil desulfinyl indicates that in vivo photodegradation may occur under field conditions and warrants further investigation regarding its contribution to residue dynamics in cattle.

## Author Contributions

**André Tadeu Gotardo:** writing – review and editing, writing – original draft, funding acquisition, project administration, conceptualization, methodology. **Giselle Kindlein** and **Silvana Lima Górniak:** writing – review and editing, writing – original draft, conceptualization, methodology. **Fabiano Barreto, Caroline Andrade Tomaszewski, Tamara dos Santos Castilhos**, and **Daniel Rodrigo Hillesheim:** methodology, formal analysis. **Stefani Maria Ferreira:** writing – review and editing, investigation, formal analysis, data curation. **Leonila Ester Reinert Raspantini:** methodology, formal analysis.

## Funding

This work was funded by the National Council for Scientific and Technological Development (CNPq), Grant N°. 404032/2023‐0.

## Ethics Statement

The procedures were approved by the Ethics Committee on the Use of Animals (CEUA) of the School of Veterinary Medicine and Animal Science, University of São Paulo (FMVZ‐USP; protocol number 7756291123).

## Conflicts of Interest

The authors declare no conflicts of interest.

## Data Availability

The data that support the findings of this study are available from the corresponding author upon reasonable request.
